# Development and validation of a prediction model for lower extremity deep vein thrombosis risk in elderly patients with intracerebral hemorrhage

**DOI:** 10.3389/fneur.2026.1815487

**Published:** 2026-07-15

**Authors:** Yi Yang, XinYi Guo, Ke Luo, Wenjuan Zhao, Hongru Li, Yongli Zhang

**Affiliations:** 1 Department of Pharmacy, Tianjin Huanhu Hospital, Tianjin Key Laboratory of Cerebral Vascular and Neurodegenerative Diseases, Tianjin, China; 2 Department of Neurology, Tianjin Huanhu Hospital, Tianjin Key Laboratory of Cerebral Vascular and Neurodegenerative Diseases, Tianjin, China; 3 Department of Pharmacy, Tianjin Medical University, Tianjin, China; 4 Department of Medical Imaging Technology, Tianjin University of Traditional Chinese Medicine, Tianjin, China

**Keywords:** D-dimer to fibrinogen ratio, deep-vein thrombosis, elderly patients, intracerebral hemorrhage, prediction model

## Abstract

**Background:**

Geriatric patients are simultaneously exposed to a high risk of both bleeding and thromboembolism, making antithrombotic management for elderly intracerebral hemorrhage patients a dilemma. This study aimed to develop and validate a prediction model for lower extremity deep vein thrombosis risk in elderly patients with intracerebral hemorrhage.

**Methods:**

Demographics variables, clinical features and laboratory parameters were retrospectively collected from elderly intracerebral hemorrhage patients at our hospital from August 2023 to July 2024. Multiple imputation was used to handle missing values. Potential predictors of lower extremity deep vein thrombosis (demographic, clinical features and laboratory parameters) were assessed using least absolute shrinkage and selection operator (LASSO) regression for feature selection followed by multivariable logistic regression, a prediction model and a nomogram were constructed. Performance of the model was assessed through the receiver operating characteristic curve (ROC), calibration curve, decision curve analysis (DCA), and clinical impact curve (CIC).

**Results:**

Two hundred and forty-seven elderly intracerebral hemorrhage patients were included in the analysis. Five potential predictors were identified after LASSO regression: logDFR, D-dimer, eGFR, GCS and infection. These predictors were subsequently entered into a multivariable logistic regression model. Highly correlated predictors were further assessed to ensure model stability and interpretability. The final model included four independent predictors: logDFR (OR 1.84, 95% CI 1.28–2.63), eGFR (OR 1.01, 95% CI 1.00–1.02), GCS (OR 0.87, 95% CI 0.79–0.96) and infection (OR 2.20, 95% CI 1.05–4.63). The model had an area under the curve (AUC) of 0.819 (95%CI 0.808–0.830), suggesting good discrimination. Internal validation was performed using bootstrap resampling, the estimated optimism was 0.028, representing expected performance. Calibration assessment indicated only moderate overestimation of risk. Decision curve analysis (DCA) and clinical impact curve (CIC) consistently demonstrated the model’s applicability across diverse clinical settings.

**Conclusion:**

This study developed and validated an effective prediction model, incorporating four clinically accessible parameters, for estimating the risk of lower extremity deep vein thrombosis in elderly patients with intracerebral hemorrhage. This model may facilitate the early identification of high-risk patients, thereby guiding prophylactic interventions.

## Introduction

Spontaneous intracerebral hemorrhage (ICH) takes on particular importance among neurologic conditions, because most patients surviving ICH suffer from disabilities and are at risk for other illnesses such as systemic vascular diseases (1). Former studies found a significantly higher rate of lower extremity deep vein thrombosis (LDVT) in patients with hemorrhagic stroke compared with thromboembolic stroke (2). This might be ascribed to a more severe neurological dysfunction and extent of paralysis in ICH (2). In addition, hemostasis procedures are activated after ICH as a way of self-protection (3). ICH begins with blood vessel rupture (4). Collagen is exposed at the site of injury, causing platelets to adhere to the damaged endothelium, leading to the formation of a platelet plug and thereby achieving primary hemostasis (5). Subsequent to platelet plug formation, secondary hemostasis is initiated. The coagulation cascade activates a series of plasma coagulation factors to complete the hemostatic process (5). These concomitant reactions may increase venous thromboembolism (VTE) risk. LDVT risk also increased with age, which may relate to an increasing occurrence of other illnesses prone to thrombosis or coagulation potential, or the combination of both (6). Moreover, geriatric patients are simultaneously exposed to a high risk of both bleeding and thromboembolism, the balance between these two risks becomes particularly delicate due to the presence of other concurrent diseases, make antithrombotic management a dilemma (7).

Most of the existing models such as Caprini and Padua are derived from data from general hospitalized patients and these models prediction of the risk of LDVT in elderly ICH patients may not be accurate (8, 9). Therefore, it is necessary to explore the risk factors of LDVT in elderly patients with ICH. Previous studies have found that besides factors included in Caprini and Padua, other factors such as D-dimer to fibrinogen ratio (DFR), Platelet to lymphocyte ratio (PLR) and dyslipidemia might also influence the risk of developing LDVT (10–12). In this study, we collected relevant data and aim to develop a prediction model of LDVT in elderly ICH patients.

## Materials and methods

### Study group

By retrospecting electronic medical records (EMR), the clinical data of 247 elderly patients with cerebral hemorrhage, hospitalized in Tianjin Huanhu Hospital from August 2023 to July 2024, were collected. Inclusion criteria included primary cerebral parenchymal hemorrhage diagnosed through CT scan within 48 h after admission, meeting the diagnostic criteria of cerebral hemorrhage (13), age of onset over 60 years old, who were not given prophylactic or therapeutic anticoagulant medication before onset of ICH or diagnosis of LDVT, and received ultrasound examination of the lower limbs blood vessels on both sides during hospitalization. Exclusion criteria included patients receiving long-term anticoagulation therapy, patients with severe underlying diseases such as severe dysfunction in cardiac, liver and renal system, patients with malignancy, and patients with previous deep venous thrombosis history or coagulation disorders. Among the cases, 82 cases developed lower limb deep vein thrombosis were include as the LDVT group. One hundred and sixty-five cases did not developed lower limb deep vein thrombosis were included as the non-LDVT group. The data of age, gender and past medical history was collected. All included patients received treatment recommended in the guideline for the management of ICH (13). IPC was initiated in all patients on the day of admission and no prophylactic anticoagulant was administered. In LDVT group, a total of 37 patients received anticoagulant therapy. Anticoagulation was initiated immediately in 24 patients on the day of LDVT diagnosis and delayed for 13 patients. Five patients underwent inferior vena cava (IVC) filter placement; among patients mentioned above, two received IVC filter concomitantly with anticoagulant therapy. The remaining 40 patients received neither anticoagulation nor IVC filter placement because of planned interhospital transfer or refusal of treatment by the patients and/or their families. Seven patients developed hematoma expansion during hospitalization, including three in the non-LDVT group and four in the LDVT group. All four hematoma expansion events in the LDVT group occurred before LDVT was diagnosed thus could not be attributed to therapeutic anticoagulation.

### Features collection

Predictor variables used in this study included baseline demographics, clinical features and laboratory parameters. Information for further analysis were collected on 24 patient features: age, gender, smoking history, alcohol consumption history, history of surgery in the past month, laboratory parameters [white blood-cell count (WBC), platelets count (PLT), lymphocyte count (LYM), procalcitonin (PCT), D-dimer (DD), fibrinogen (FIB), albumin (ALB), serum creatinine and serum lipid levels] and comorbidities [hypertension, diabetes mellitus, infection (including pulmonary infection and urinary infection)]. Hypoalbuminemia was defined as serum values of <3.5 g/dL (14). PLR was calculated from the value of PLT divided by the value of LYM. DFR was calculated from the value of DD divided by the value of FIB. The normal reference value of DD: 0.01–0.5 mg/L, FIB: 2–4 g/L. Estimated glomerular filtration rate (eGFR) was calculated from serum creatinine and age using the abbreviated MDRD equation for Chinese patients (15). The patients’ level of consciousness was calculated using Glasgow coma scale (GCS) (16). The risk stratification of ICH was conducted with the ICH score (17).

Ultrasound examination was conducted after admission for all patients included within 7 days, if the initial result was negative, the examination was repeated when the clinicians suspected LDVT occurrence until the patients were discharged. Nineteen patients ended up with a positive subsequent result were transferred to LDVT group. All patients in LDVT group had positive results while all patients in non-LDVT group had only negative results. Serum samples were acquired on the first day of admission, then measured in the clinical laboratory of our hospital. The value of WBC, PLT and LYM were determined using hematological analyzer system on the Sysmex XN-1000 (Sysmex Company, Japanese). The value of DD, FIB, ALB, serum creatinine and serum lipid levels were determined using clinical chemistry assays on the Abbott c8000 system (Abbott Diagnostics, USA). The value of PCT was determined using chemiluminescence immunoassay on the Cobas E411 (Roche Diagnostics, USA). The diagnosis of LDVT was based on the assessment results provided by radiologists. Missing values were taken care of using multiple imputation.

### Statistical processing

R Statistical Software (version 4.5.1)^1^ was used for statistical analysis. There were some missing values in smoking history, alcohol consumption history, ICH volume, PCT, DD, FIB, hypoalbuminemia, serum creatinine, TG, TC, LDL-C and HDL-C, after confirmation of missing at random (MAR), we multiply-imputed the missing data using MI based on the Predictive Mean Matching (PMM). Since age and all laboratory parameters used in this study have a logical lower bound as zero while other patient features have binary values, PMM is a suitable MI algorithm for our study, for only plausible values are imputed for the missing data, allowing distributions to be preserved (18). The number of iterations was set to 5 and the number of multiple imputation was set to 40, i.e., *M* = 40 (19).

Normally distributed continuous variables were described in the form of mean ± standard deviation (SD), normality was assessed separately in each group using skewness statistics. Variables showing approximate normality were compared using t-tests (Welch correction when appropriate). For variables with non-normal distributions in either group, data were summarized as median (interquartile range) and compared using the Mann–Whitney U test. Categorical data were presented as counts (percentage), with statistical significance evaluated with chi-square. Assumption of linearity of independent variables was assessed by Box-Tidwell transformation. For variables that were nolinear and right-skewed, log-transformation was performed. As the variables included ordinal data and some continuous variables exhibited non-normal distribution, Spearman’s rank correlation analysis was used to assess correlations between potential independent risk factors, for it is a nonparametric statistical technique. The correlation structure was visualized using a heatmap. LASSO regression was applied to identify potential predictors. Selected predictors were further incorporated into a multivariate logistic regression to construct a predictive model. The model’s discriminative performance was assessed using the area under the curve (AUC) of the receiver operating characteristic curve (ROC), calibrated performance was assessed with internal validation through 1,000 bootstrap iterations. Clinical usefulness was examined using decision curve analysis (DCA) and clinical impact curves (CIC).

## Results

### Study patients characteristics

**Figure 1:**
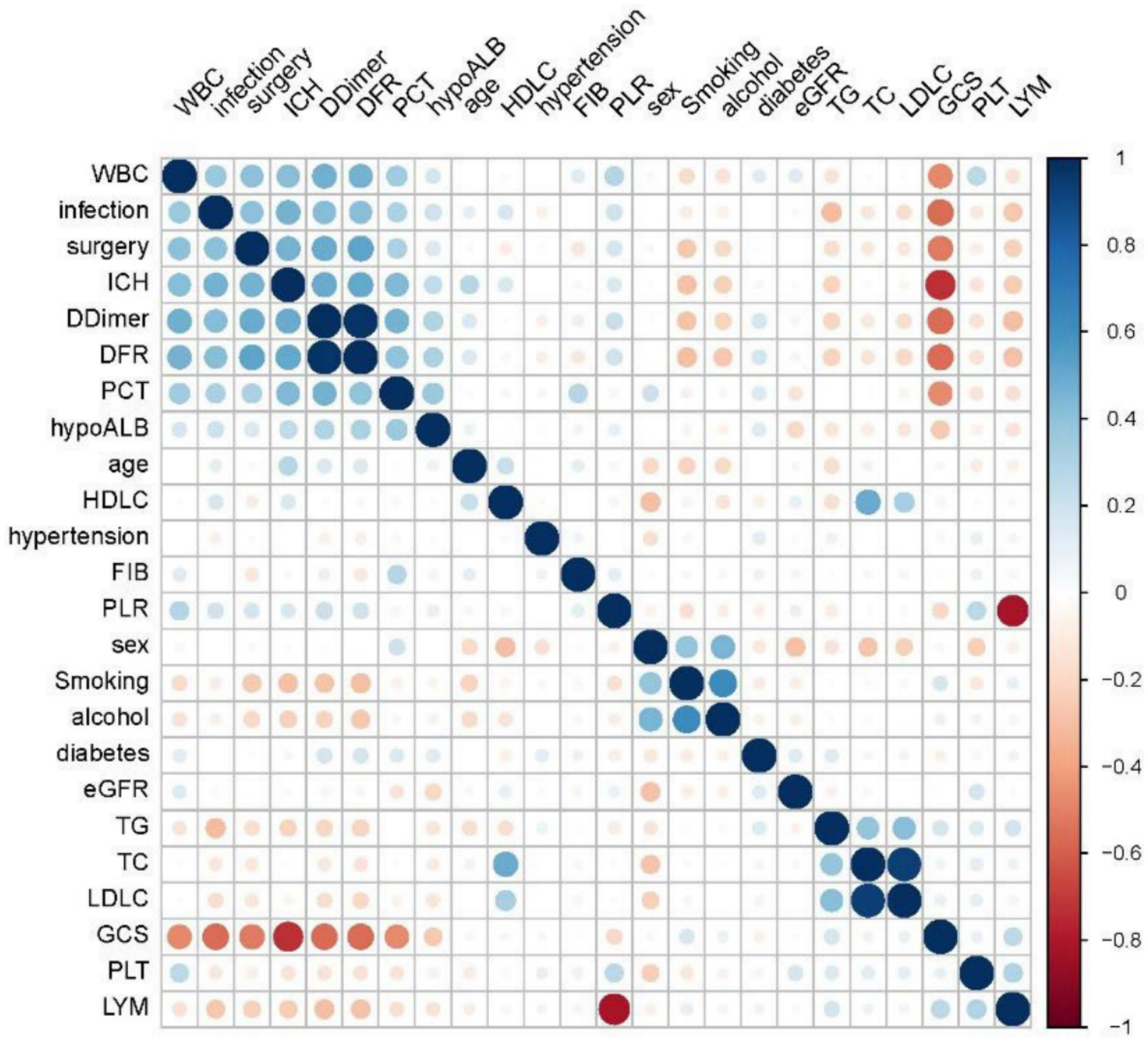
Correlation matrix between potential independent risk factors. The color and area of the circles in the matrix reflects the correlation between the variables. Color blue indicated positive correlation while color red indicated negative correlation. The larger the area, the darker the color, the stronger the correlation between variables.

Table 1 summarized the baseline demographic, clinical characteristics and laboratory parameters for patients with and without LDVT. A total of 247 patients were included, 82 of them were in the LDVT group and the rest 165 patients were in the non-LDVT group. Among all cases, 107 patients (43.32%) were female, with a mean age of 70.71 years. Between these two groups, there was no statistic differences in gender, age, history of smoking or alcohol drinking, past-history of hypertension, diabetes, PLT, FIB, TG, TC, LDL-C, HDL-C. While differences in GCS, ICH score, infection, surgery, WBC, LYM, PCT, DD, PLR, DFR, hypoalbuminemia and eGFR were statistically significant. The multiple imputation further confirmed the robustness of these results (Supplementary Table S1). Sensitivity analysis confirmed model robustness, with approximate AUC (0.862 vs. 0.819) (Supplementary Figure S1). Spearman’s rank correlation analysis was used to assess correlations between variables (Figure 1). According to the result of the Box-Tidwell test (Supplementary Table S2), we performed log-transformation on LYM, PCT, PLR and DFR.

**Table 1:** Baseline demographic and clinical characteristics, laboratory parameters of the patients.

Variables	Non-LDVT group (165)	LDVT group (82)	t/*Χ^2^*	*p* value
Sex (female, %)	69 (41.82%)	38 (46.34%)	0.456	0.499
Age (year)	70.43 ± 7.00	71.27 ± 6.46	0.909	0.364
Smoking (*n*, %)	58 (35.58%)	27 (32.93%)	0.170	0.680
Alcohol (*n*, %)	43 (26.38%)	19 (23.17%)	0.297	0.586
Hypertension (*n*, %)	143 (86.67%)	71 (86.59%)	<0.001	0.986
Diabetes (*n*, %)	32 (19.39%)	19 (23.17%)	0.477	0.490
GCS	12.46 ± 3.17	9.27 ± 3.98	−6.337	<0.001*
ICH score	1.43 ± 1.14	1.90 ± 1.12	2.972	0.003*
Infection (*n*, %)	59 (35.76%)	60 (73.17%)	30.710	<0.001*
Surgery (*n*, %)	30 (18.18%)	34 (41.46%)	15.467	<0.001*
WBC (×10^9^)	8.39 ± 3.12	10.45 ± 3.51	4.501	<0.001*
PLT (×10^9^)	209.00 (76.00)	199.00 (73.50)	–	0.433
LYM (×10^9^)	1.31 (0.91)	1.09 (0.62)	–	0.004*
PCT (ng/ml)	0.05 (0.06)	0.07 (0.17)	–	0.002*
DD (mg/L FEU)	0.76 (1.33)	3.50 (8.27)	–	<0.001*
FIB (g/L)	2.94 (0.96)	2.91 (1.41)	–	0.783
PLR	157.60 (119.57)	198.05 (118.89)	–	0.008*
DFR	0.24 (0.37)	1.06 (3.07)	–	<0.001*
Hypoalbuminemia (*n*, %)	15 (9.20%)	17 (20.73%)	6.288	0.012*
eGFR (ml/min/1.73m^2^)	100.98 (35.51)	118.73 (40.88)	–	0.017*
TG (mmol/L)	1.16 (0.78)	0.98 (0.74)	–	0.123
TC (mmol/L)	4.58 ± 1.22	4.61 ± 1.23	0.163	0.871
LDL-C (mmol/L)	2.81 ± 0.88	2.78 ± 0.92	−0.220	0.826
HDL-C (mmol/L)	1.23 ± 0.28	1.30 ± 0.32	1.509	0.134

*p < 0.05.

### Construction, validation and performance of the prediction model

**Figure 2:**
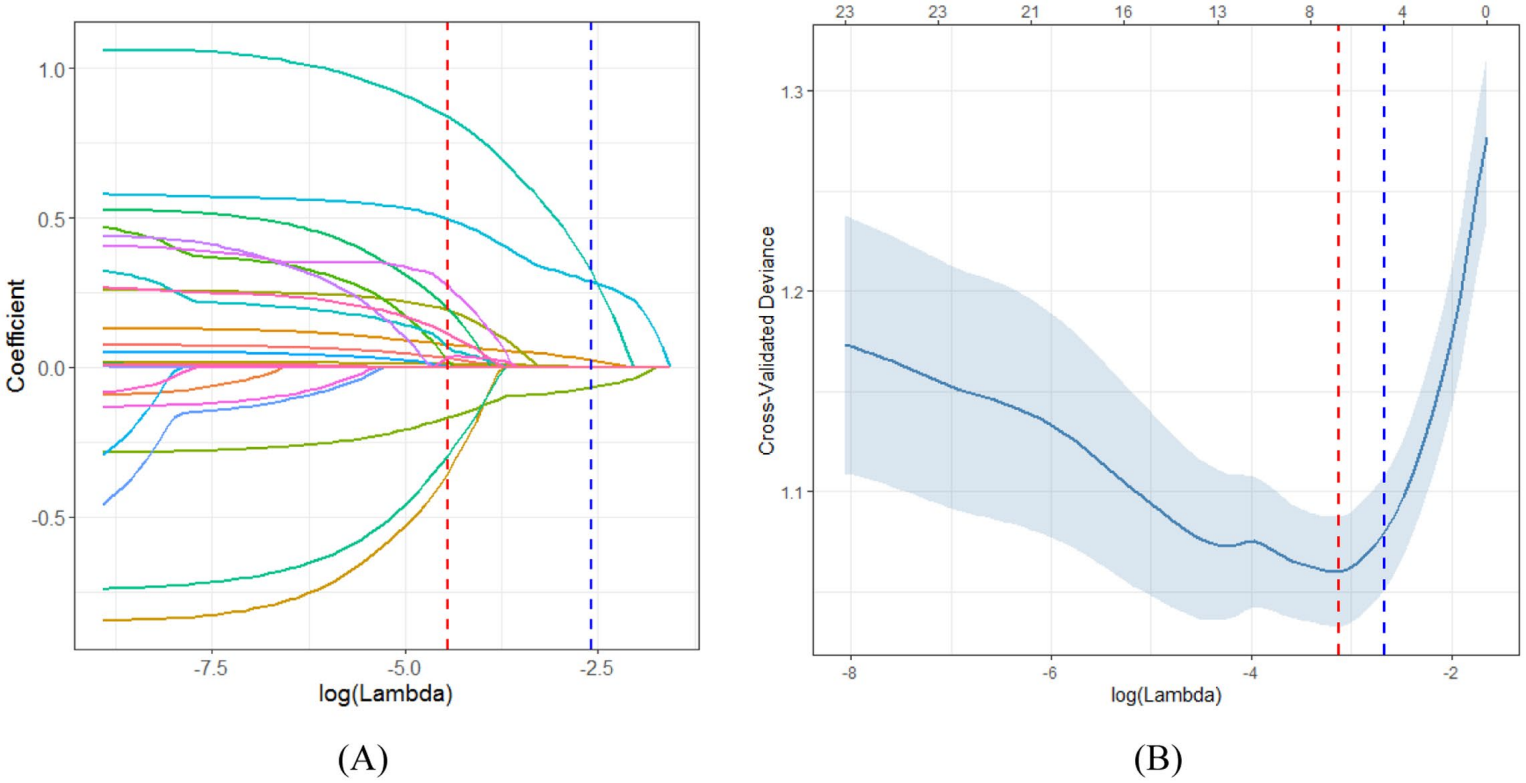
Graphical illustration of the shrinkage process. **(A)** Coefficient profile plot: LASSO coefficients of 24 features (y-axis) vs. log (*λ*) (x-axis); blue vertical dashed line indicates λ_1_se. **(B)** Binomial deviance plot: Mean deviance (y-axis) vs. log (λ) (x-axis); blue vertical dashed line marks λ₁se from 10-fold cross-validation.

Variable selection was performed using LASSO regression within each imputed dataset. Ten-fold cross-validation was used to determine the optimal penalty parameter (*λ*), and the λ corresponding to the one–standard-error rule (λ_1_se) was selected to improve model parsimony and stability. Predictors selected in a majority of the imputed datasets were considered stable and retained for subsequent model development. Twenty-four candidate variables were screened and 5 predictive factors with non-zero coefficients were identified: DD, logDFR, GCS, eGFR and infection (Figures 2A,B).

**Figure 3:**
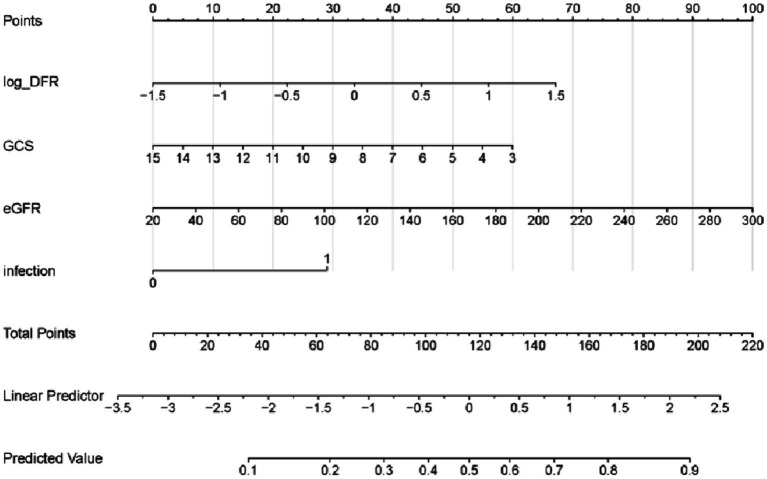
Nomogram for predicting LDVT risk in elderly ICH patients: variables assigned scores; total score estimates LDVT occurrence probability.

**Figure 4:**
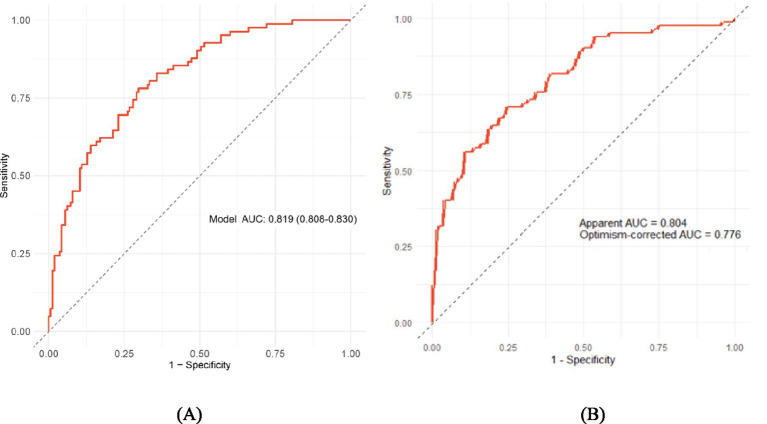
ROC curve and AUC: **(A)** Apparent discriminative performance of the model. **(B)** Internal calibrated discriminative performance of the model.

The result of correlation analysis indicated that DD and DFR were highly collinear and DFR was a derived indicator of DD, thus the value of logDFR and DD could also be correlated, only one of the two variables would be retained in the final multivariable model to avoid multicollinearity. Therefore DD and logDFR were included into multivariable logistic regression separately, yielded two models: Model A and Model B. Model A included logDFR, eGFR, GCS and infection. Model B included DD, eGFR, GCS and infection. The AUC of Model A was 0.819 (95%CI 0.808–0.830) with a sensitivity of 79.18% and specificity of 70.03%. Model B had an AUC of 0.827 (95%CI 0.814–0.839) exhibiting a sensitivity of 88.14% and specificity of 61.11%. The pooled difference in AUC between the two models was −0.008 (95% CI −0.027 to 0.012; *p* = 0.45), indicating no statistically significant difference in discriminative ability. Given the potential bleeding risks associated with anticoagulation therapy in elderly patients, clinical decision-making often favors a good-specificity strategy to avoid unnecessary treatment. While Model B had better sensitivity, its specificity was relatively poor. Model A demonstrated fair sensitivity and specificity, therefore we chose Model A as the final model. Predictors in the final model were logDFR (OR 1.84, 95% CI 1.28–2.63, *p* = 0.001), eGFR (OR 1.01, 95% CI 1.00–1.02, *p* = 0.027), GCS (OR 0.87, 95% CI 0.79–0.96, *p* = 0.008) and infection (OR 2.20, 95% CI 1.05–4.63, *p* = 0.038). The full model equation was: logit(*P*) = −0.397 + 0.608*logDFR + 0.010*eGFR − 0.136*GCS + 0.788*infection, where logDFR was natural logarithm of DFR, eGFR was estimated glomerular filtration rate (ml/min/1.73m^2^), GCS was Glasgow Coma Scale score and infection was acute infectious diseases (pulmonary infection or urinary infection, 1 if present, 0 if absent). An optimal cutoff of 0.492 was identified, determined by the Youden index. The nomogram was showed in Figure 3. The apparent discriminative performance of the model yielded an AUC of 0.819 (95% CI: 0.808–0.830) (Figure 4A).

**Figure 5:**
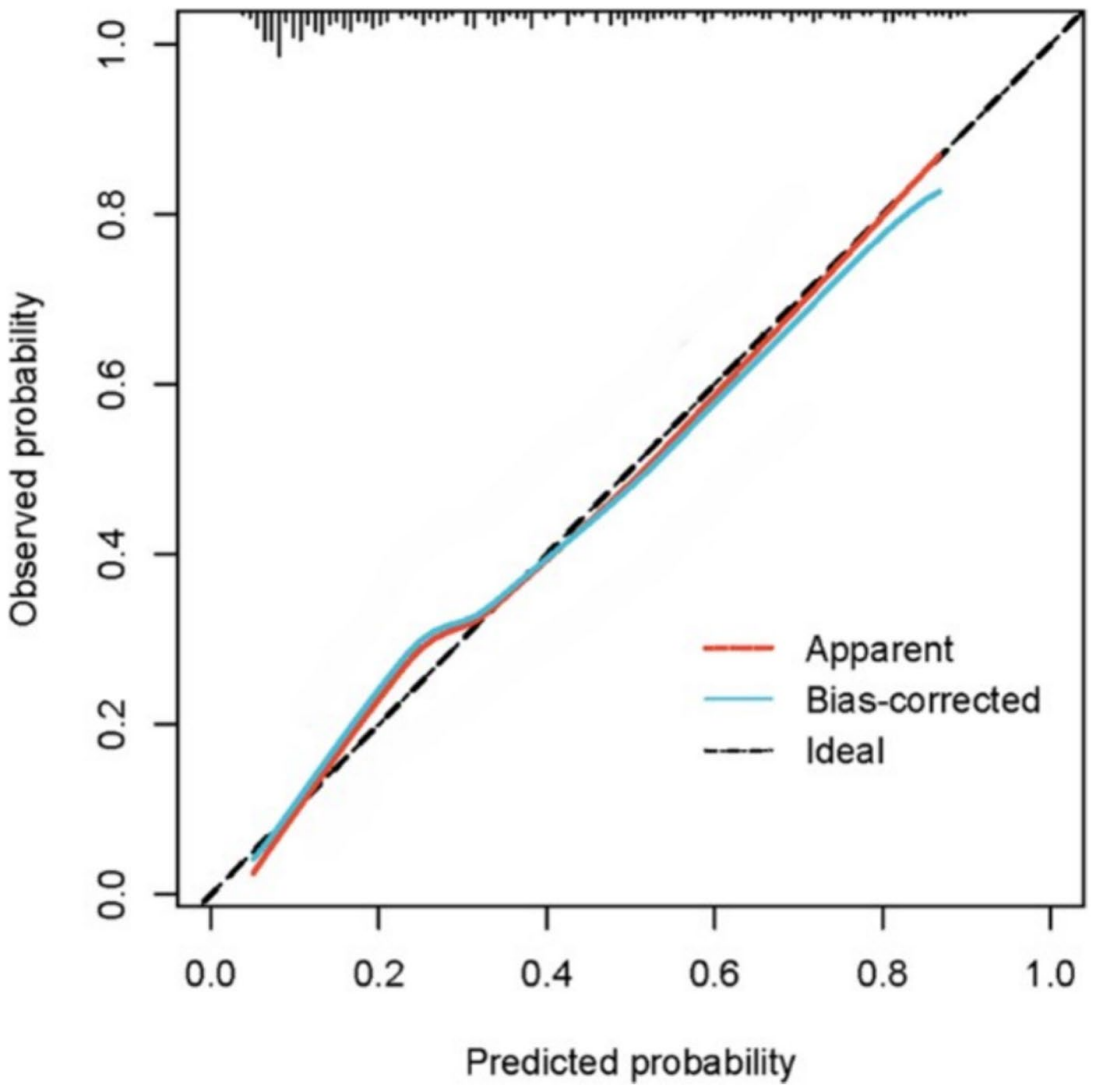
Calibration curves of the logistic regression models. The red line represents the apparent calibration, the blue line represents the bootstrap bias-corrected calibration, and the dash line indicates ideal calibration. Optimism-corrected Calibration Intercept: −0.0276 (95% CI: −0.0842–0.0291), optimism-corrected Calibration Slope: 0.936 (95% CI: 0.809–1.0624).

Internal validation was performed using bootstrap resampling (1,000 times) with the entire modeling process, including variable selection, repeated in each resample. The bootstrap apparent AUC was 0.804, with an estimated optimism of 0.028. After optimism correction, the AUC was 0.776, indicating good discriminative ability (Figure 4B). The calibration plot demonstrated satisfactory agreement between predicted and observed risks (Figure 5).

**Figure 6:**
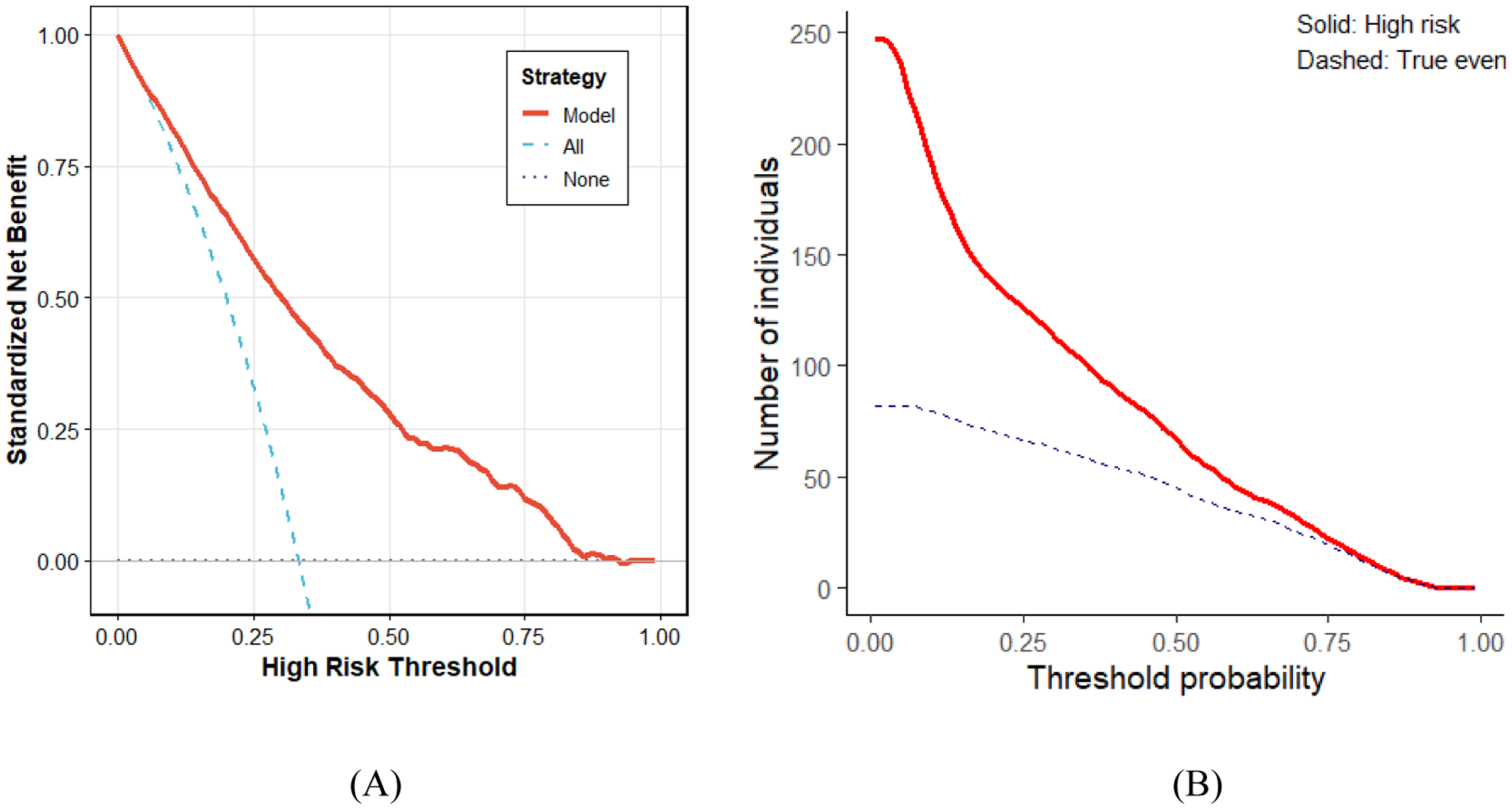
**(A)** DCA of the model. Within a risk threshold range of 5 to 88%, the net clinical benefit of the model was superior to both the “treat all” and “treat none” strategies. **(B)** CIC of the model. As the high-risk threshold increased, both the number of patients classified as high-risk by the model (solid red line) and the actual number of LDVT events (dashed blue line) exhibited a decreasing trend. Substantial overlap was observed at up to approximately 80% threshold, indicating minimized over-prediction.

DCA (Figure 6A) was employed to illustrate the net clinical benefit of the model across various risk thresholds. Furthermore, CIC (Figure 6B) was used to reflect the model’s efficacy in stratifying and identifying high-risk patients. The findings of this study confirmed that the predictive model possessed reliable clinical utility for predicting the occurrence of LDVT in elderly patients with ICH.

## Discussion

LDVT is a common complication in ICH patients. Predicting the risk of LDVT in elderly patients with ICH is particularly important, because the possibilities of thrombosis and hemorrhage in elderly patients are both relatively high, which would be difficult to balance during treatment. In our study, we constructed a prediction model for LDVT occurrence in elderly patients with ICH. The model had balanced performance, with good sensitivity and specificity. The DCA in our study demonstrated that the proposed prediction model provides meaningful clinical utility across a wide range of threshold probabilities. Moreover, the CIC further revealed that at higher threshold probabilities, the number of patients identified as high risk closely approximated the number of observed LDVT cases, suggesting minimal overestimation and a high positive predictive value in this range. In this setting, our findings suggested that the model is suitable for guiding prophylactic anticoagulation initiation under a high-threshold strategy. By restricting treatment to patients with substantially elevated predicted risk, clinicians may reduce avoidable complications such as bleeding while maintaining overall clinical benefit. Although some predictors included in this model have also been identified as risk factors for VTE in other conditions (20), the model was specifically derived from elderly patients with ICH. ICH involves unique pathophysiological mechanisms, including vascular rupture, hematoma-induced mass effect, and protective activation of the coagulation cascade (6). Given the differences in underlying pathophysiological mechanisms and baseline thrombotic risk across ICH patients with general ICU populations and patients with other CNS injuries, our model’s performance might not be directly applicable to other clinical populations. Accordingly, the model should not be applied outside the elderly ICH population without further validation.

The four independent risk factors included in the model were logDFR, infection, GCS and eGFR. These factors collectively reflect the multi-factorial pathogenic mechanism of LDVT, including coagulation cascade activation, systemic inflammation, hemodynamics and physiological characteristics of elderly patients.

The original design purpose of DFR was to increase the specificity of diagnosing patients with pulmonary embolism (21). These results have been repeatedly verified in subsequent studies on patients with pulmonary embolism (22, 23). The activated coagulation process leads to the consumption of FIB in the pulmonary vasculature, while the activation of the fibrinolysis process causes an increase in the level of DD (21). Therefore DFR level as the ratio of DD and FIB appeared to raise. However, the variation trend of DFR was different in patients with ICH. In this study, FIB was slightly but not statistically significantly lower in the LDVT group than in the non-LDVT group, the decrease in FIB due to thrombosis did not seem to be apparent. We supposed this might be connected to the function of FIB as an inflammatory marker (24). After ICH, inflammatory pathological changes occur, involving related processes within the brain as well as circulating blood (25). As a result, although FIB is consumed during the activation of the coagulation cascade, it is also produced by liver cells during inflammatory responses as an acute-phase reactant (26). Due to these opposite effects, FIB shows a poor diagnostic ability for LDVT in patients with ICH.

In previous studies, DD was found to be related to thrombosis events. It can only be produced upon formation and degradation of cross-linked fibrin, therefore it can comprehensively reflect the activation status of the coagulation and fibrinolysis system, and also serve as an indirect indicator of thrombosis activity (27). Due to its negative predictive value, it is often used to rule out the diagnosis of venous thrombosis. However, the mere increase in DD concentration alone cannot confirm the diagnosis of DVT, nor can it be used for positive prediction, as an increase in DD may also occur in patients with fracture, malignant tumor, trauma, recent surgery and infections (28). DD values also increase with age, the use of age-adjusted cutoff values for DD tests increase its specificity in the elderly population (29). In our study the age of the elderly patients included in both groups was similar, so there should be no unwanted influence of advanced age on the predictor variables. DFR, as a composite index, is less affected by factors such as inflammation and can reflects the dynamic equilibrium state of the coagulation-fibrinolysis system more stably (30). A limited number of studies have addressed the prediction value of DFR at the risk of VTE (31–33). Wen et al. (10) discovered that DFR combined with Wells score have high specificity for predicting LDVT in young patients with cerebral hemorrhage. Yet in these studies, elderly individuals were either excluded from the study or were only part of the study subjects. None or very limited subgroup analyses were mentioned, and the sample size of older patients was too small to conduct meaningful analyses.

The infectious diseases referred in this study included pulmonary infections and urinary tract infections. Epidemiological researches have indicated that respiratory infections were associated with increasing risks of vascular diseases, including venous thrombosis. One potential mechanism was the occurrence of coagulation and platelet activation during pneumonia, which might induce thrombogenesis within venous circulation (34). Another study has revealed that acute lower respiratory tract infection elevated the risk of cardiovascular events in elderly patients with stable coronary artery disease, which might be attributed to platelet-mediated inflammatory responses that could precipitate or exacerbate vascular injury and dysfunction. Furthermore, bacteria and their byproducts are capable of activating and aggregating platelets. Concurrently, circulating inflammatory mediators or direct vascular endothelial infection by the pneumonic pathogen could lead to endothelial damage, disrupting the homeostatic balance between the coagulation and fibrinolytic systems, and consequently contribute to the pathogenesis of ischemic diseases (35). After urinary infection, the risk of DVT and pulmonary embolism was also significantly elevated. This effect is similar to what has been observed after respiratory infection, suggesting the influence of infection on the risk of VTE might be a general phenomenon, rather than being specific to a particular type of infection (36).

GCS has been extensively utilized in various clinical settings to assess the level of consciousness, particularly for evaluating arousability and awareness (37). It was incorporated into ICH score which estimates the risk of death and functional outcome for ICH patients. In former study, the GCS scores of ICH patients with VTE are significantly lower than those without VTE (2), consistent with the result of our research. Paresis/paralysis and reduced mobility are known risk factors of DVT (38). Consciousness impairment can decrease mobility of patients, reduce muscle contraction and venous return of lower limbs, thus promotes lower extremity thrombosis (39, 40).

In this study, the median eGFR values were relatively high in both the LDVT and non-LDVT groups. However, the accuracy of the eGFR formula is suboptimal within higher range, exhibiting poor correlation with renal function (15). The elevation in eGFR observed in this study was associated with the decrease in serum creatinine levels. Serum creatinine is a derivative of skeletal muscle protein metabolism, its rate of production varies in accordance with changes in skeletal muscle mass (41). Serum creatinine concentration reflects the equilibrium between muscle mass and renal excretory function (42). Our results suggested that elderly patients in the LDVT group were more likely to have reduced muscle mass and sarcopenia was considered a biological basis for frailty (43). In patients undergoing hemodialysis, those identified as frail exhibited a higher risk of vascular access thrombosis compared to non-frail individuals (44). For SAH patients, frailty was associated with increased risk of DVT (45). Platelet function was known to increase with advancing age. Furthermore, within the elderly population, a higher degree of platelet activation has been observed in the frailty group (46). This mechanism might also contribute to the pathogenesis of LDVT.

### Limitations

Our study had some limitations need to be discussed. The data were obtained through a retrospective method, susceptible to selection bias, and the model was not externally validated. These limitations may reduce statistical power and inflate the influence of random variation. We addressed these limitations through correlation analysis and internal validation. This study employed multiple imputation to handle missing values. This approach relies on the MAR assumption, which states that missingness can be fully explained by observed variables. Despite the assessment of missing data patterns, the presence of a missing not at random (MNAR) mechanism cannot be completely excluded. Patients included in this study are all Chinese and the abbreviated MDRD equation we used was modified for Chinese patients. If the prediction model of our study would be applied to other population, further modification might be required. The follow up of our study was limited to the hospital stay, no follow up was scheduled after discharge. It was possible that cases of LDVT occurred after discharge have been omitted. This needs to be improved in future researches. Notably, although lower threshold probabilities may increase sensitivity, they may also lead to overtreatment. Therefore, the model should not primarily be regarded as a screening tool, but rather as a decision-support instrument for identifying patients who are sufficiently high risk to justify therapeutic intervention. Future prospective validation studies are warranted to determine the optimal clinically acceptable threshold and to evaluate the real-world impact of model-guided anticoagulation strategies.

## Conclusion

Several studies have explored predictive models for DVT, but only a limited number of researches have addressed ICH patients, and these studies were primarily focused on younger populations. Older patients with ICH are more prone to develop DVT. Thus establishing a scientific risk prediction system hold significant practical value. In this study, we have developed a model to predict the risk of LDVT for elderly patients with ICH. Four factors were integrated in the model: (1) natural logarithm of DFR, (2) estimated glomerular filtration rate (ml/min/1.73m^2^), (3) Glasgow Coma Scale score and (4) acute infectious diseases. This model could serve as a decision-supporting tool for identifying high-risk patients, enabling personalized management and early prevention.

## Data Availability

The raw data supporting the conclusions of this article will be made available by the authors, without undue reservation.

## Footnotes

1
https://www.r-project.org
