# Supplementary material for: Development and validation of a prediction model for lower extremity deep vein thrombosis risk in elderly patients with intracerebral hemorrhage
Source: Front Neurol. 2026 Jul 15;17:1815487. doi: 10.3389/fneur.2026.1815487 (PMC13414270; doi:10.3389/fneur.2026.1815487)
Supplement: Supplementary file 1 [file Data_Sheet_1.docx]

Table S1 Patients characteristics with missing values before and after multiple imputation

| Variables | Number(%) with missing data | complete case  [Mean±sd/Median(IQR)/ %] | multiple imputed data  [Mean±sd/Median(IQR)/ %] |
| --- | --- | --- | --- |
| Smoking (n, %) | 0.81 | 34.69% | 34.87% |
| Alcohol (n, %) | 0.81 | 25.31% | 25.64% |
| ICH | 3.64 | 1.58±1.15 | 1.58±1.16 |
| PCT(ng/ml) | 37.25 | 0.06(0.08) | 0.05(0.07) |
| DD (mg/L FEU) | 19.43 | 1.32(3.13) | 1.21(2.73) |
| FIB (g/L) | 2.43 | 2.93(1.03) | 2.93(1.03) |
| DFR | 21.86 | 0.38(0.96) | 0.39(0.92) |
| Hypoalbuminemia (n, %) | 0.81 | 13.06% | 13.05% |
| eGFR (ml/min/1.73 m^2^) | 10.12 | 109.90(42.79) | 106.53(43.46) |
| TG(mmol/L) | 15.38 | 1.11(0.80) | 1.12(0.81) |
| TC(mmol/L) | 14.98 | 4.59±1.22 | 4.59±1.22 |
| LDL-C(mmol/L) | 14.98 | 2.80±0.89 | 2.80±0.89 |
| HDL-C(mmol/L) | 15.38 | 1.26±0.30 | 1.26±0.30 |


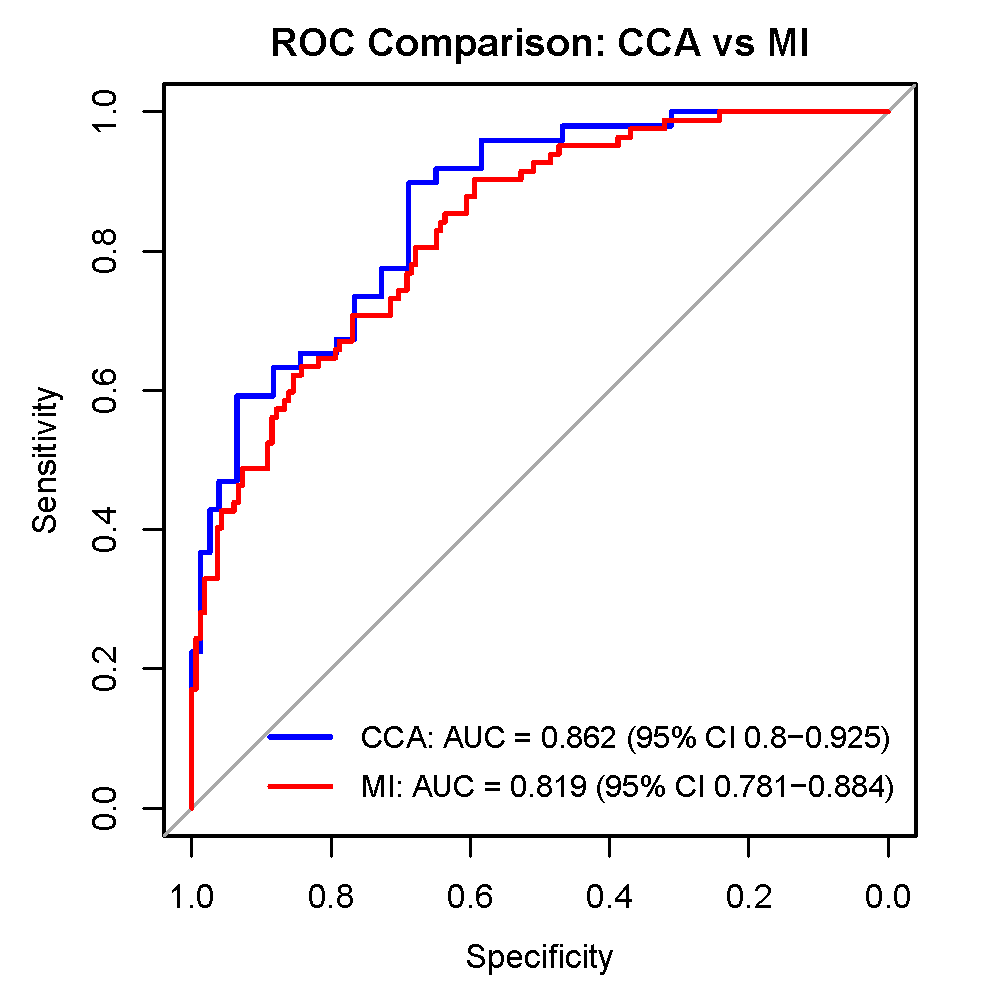


Figure S1. ROC comparison between complete case (CCA) and multiple imputed data (MI).

Table S2 Result of the Box-Tidwell test

| Variables | original Box-Tidwell *p* value |
| --- | --- |
| age | 0.205 |
| GCS | 0.103 |
| ICH_score | 0.060 |
| PLT | 0.893 |
| WBC | 0.294 |
| LYM | 0.031* |
| PCT | 0.018* |
| DDimer | 0.094 |
| FIB | 0.093 |
| eGFR | 0.170 |
| TC | 0.906 |
| TG | 0.310 |
| LDLC | 0.722 |
| HDLC | 0.519 |
| PLR | 0.019* |
| DFR | 0.005* |

**p*<0.05 indicated a significant deviation from linearity, suggesting a non-linear relationship with the logit of the outcome. Therefore, these variables were log-transformed before inclusion in the multivariable model.
